# *Situs ambigus* avec rate baladeuse et mésentère commun découvert grâce à l’imagerie médicale à Niamey (Niger): à propos d’un cas

**DOI:** 10.11604/pamj.2022.43.144.32397

**Published:** 2022-11-17

**Authors:** Inoussa Daouda Bako, Hissene Mahamat, Habiba Tinao, Matallah Sako, Malika Nababa, Yasmina Djiga, Nana Mariama Roufai, Nana Bassira Issa, Fatimata Amatkoul, Cheffou Sadi, Ibrahim Habou, Aminou Oumarou, Alexandre Akpovi

**Affiliations:** 1 Faculté des Sciences de la Santé, Université Abdou Moumouni de Niamey, Niamey, Niger,; 2 Service de Radiologie de l´Hôpital Général de Référence de Niamey, Niamey, Niger

**Keywords:** *Situs ambigus*, mésentère commun, rate baladeuse, malformation, cas clinique, Situs inversus, common mesentery, swollen spleen, malformation, case report

## Abstract

Le situs ambigus est une anomalie rare de positionnement des organes thoraco abdominaux pouvant être associé à plusieurs autres malformations dont le mésentère commun qui est une anomalie rare de rotation du tube digestif et la rate baladeuse qui est une anomalie de position de la rate, entité rare le plus souvent décrite chez les enfants. Nous rapportons ici une association de 3 entités rares chez un patient de sexe masculin âgé de huit (8 ans) admis pour le bilan échographique d´une douleur abdominale chronique. L´examen clinique était marqué par la présence d´une masse pelvienne sensible. L´échographie avait retrouvé une anomalie de position du foie et de la rate (le foie dans l´hypochondre gauche et la rate dans l´hypochondre droit) et une masse ovalaire d´écho structure tissulaire iso échogènes à la rate, de siège pelvien supra vésical, latéralisée à droite. Au scanner, il a été plus clairement établi que: le foie occupait les deux hypochondres avec le hile et la vésicule biliaire sur la ligne médiane; la loge splénique était vide et la masse pelvienne sus vésicale était homogène, iso dense au parenchyme splénique et se rehaussait de façon identique à la rate (il s´agissait d´une rate flottante), son pédicule vasculaire artériel était directement relié à l´aorte abdominale. Une anomalie de rotation du tube digestif à type de mésentère commun complet (de type habituel) a été noté.

## Abstract (en)

Situs inversus is a rare anomaly characterized by mirror-image location of of thoracic and abdominal organs. It can be associated with several other malformations including common mesentery which is a rare type of malrotation that develops as a result of the rotation of the intestines and with wandering spleen which is a rare anomaly characterized by the unusual position of the spleen in the abdomen and that most often occurs in children. We here report the case of a male patient aged 8 years diagnosed with these three entities. He was admitted with chronic abdominal pain. Ultrasound assessment was performed. Clinical examination showed sensitive pelvic mass. Ultrasound objectified abnormal position of the liver and spleen (the liver in the left hypochondrium and the spleen in the right hypochondrium) and an oval isoechogenic mass characterized by tissue structures within the echo image in the spleen, located in the supravesical fossa, lateralized to the right. CT scan confirmed that the liver occupied both hypochondria, with the hilus and gallbladder found on the midline; the splenic fossa was empty and the supra-vesical pelvic mass was homogeneous, isodense to splenic parenchyma and raised in the same way to the spleen (it was a floating spleen), its arterial vascular pedicle was directly connected to the abdominal aorta. Intestinal rotation abnormality suggesting common mesentery was also objectified.

## Introduction

Le *situs ambigus* (isomérisme) est une anomalie de la latéralisation durant l'embryogénèse aboutissant à une anatomie atypique. Il s´agit d´une configuration intermédiaire entre le situs inversus et le *situs solitus* avec un ou plusieurs organes en symétrie voire dupliqués. Il est défini par la symétrie de certains viscères par rapport au plan sagittal: dextro-isomérisme ou isomérisme droit quand les 2 moitiés droite et gauche ont la morphologie de la moitié droite normale (cas d´asplénie); lévo-isomérisme ou isomérisme gauche dans le cas contraire (polysplénie) [1]. Ainsi l´isomérisme droit est un dédoublement du côté droit; la configuration anatomique thoraco-abdominale des côtés droit et gauche étant identiques à l'image du côté droit par rapport à l´axe du corps, on a donc une asplénie: duplication des organes situés à droite dans le *situs solitus* et absence possible des organes situés à gauche. Le mésentère commun est une anomalie rare de rotation du tube digestif. Nous apportons un cas particulier d´isomérisme droit avec présence d´une rate baladeuse sus vésicale associant un mésentère commun complet découvert à l´échographie puis confirmé par la tomodensitométrie.

## Patient et observation

**Information sur le patient**: il s´agit d´un patient âgé de huit (8) ans au moment du diagnostic, de sexe masculin, sans antécédent médical ni chirurgical particulier, admis pour le bilan échographique d´une douleur abdominale évoluant depuis plusieurs mois.

**Résultats cliniques**: l´examen clinique retrouvait au niveau du pelvis une masse palpable, mobile, sensible d´environ 40 mm de diamètre.

**Figure 1:**
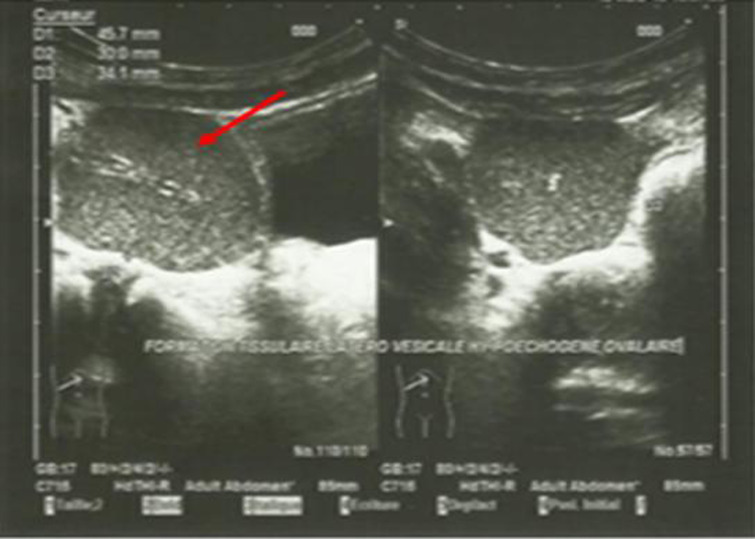
Image échographique montrant une masse ovalaire sus-vésicale d’échostructure tissulaire iso échogènes à la rate

**Démarche diagnostique**: l´examen d´imagerie médicale demandé a d´abord été une échographie abdomino-pelvienne. Elle a été réalisée avec un appareil de marque Mindray DC70 mis en service pour la première fois le 2020. Une sonde abdominale multifréquence de 2>.5- 5 Mhz a été utilisée. L´examen a été réalisé chez un patient dévêtu, en décubitus dorsal. L´échographie avait montré une anomalie de position du foie et de la rate: le foie dans l´hypochondre gauche et la rate dans l´hypochondre droit (Figure 1). Une hépatomégalie homogène avec un grand axe estimé à 132 mm; une rate de petite taille dont le grand axe est mesuré à 32 mm. Il est également retrouvé une masse ovalaire d´écho structure tissulaire iso échogène à la rate, de siège pelvien supra vésical, latéralisée à droite avec un grand axe estimé à 45,7 mm (Figure 1).

**Figure 2:**
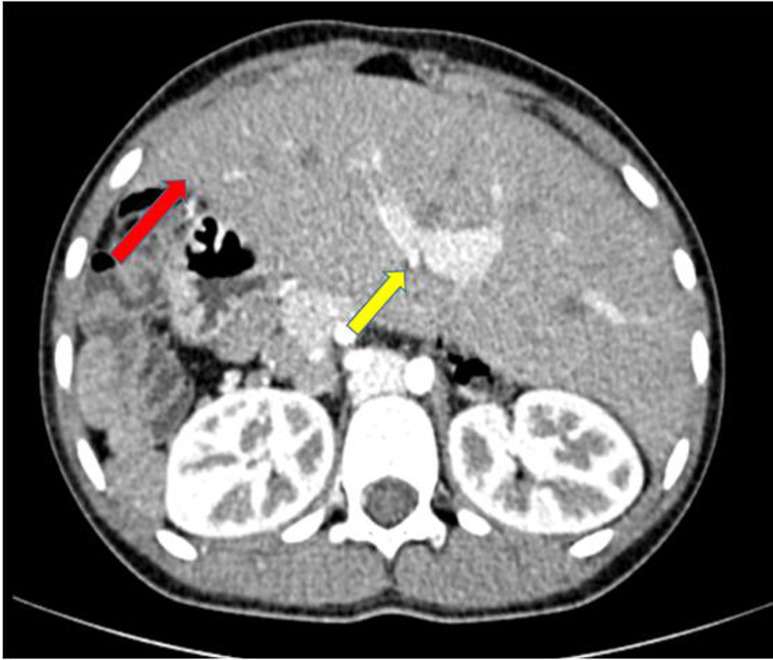
TDM abdominale en coupe axiale à hauteur du foie, après injection de PDC, montrant un foie occupant les 2 hypochondres droit et gauche (flèche rouge) et le hile du foie sur la ligne médiane (flèche jaune)

**Figure 3:**
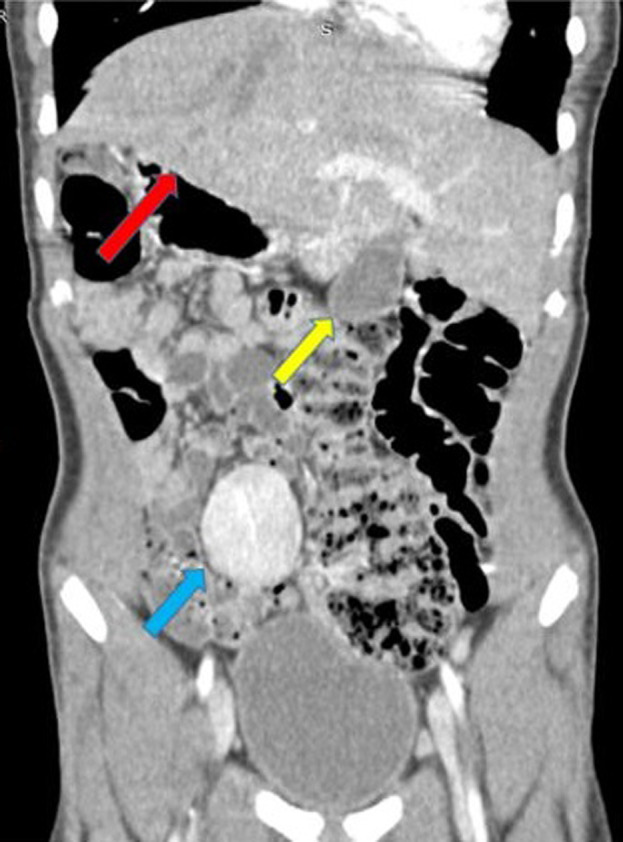
TDM abdominale en coupe coronale après injection de PDC montrant une rate baladeuse sus vésicale (flèche bleue), le foie dans les deux hypochondres (flèche rouge), la vésicule biliaire médiane (flèche jaune), pointe du cœur à gauche (flèche verte)

**Figure 4:**
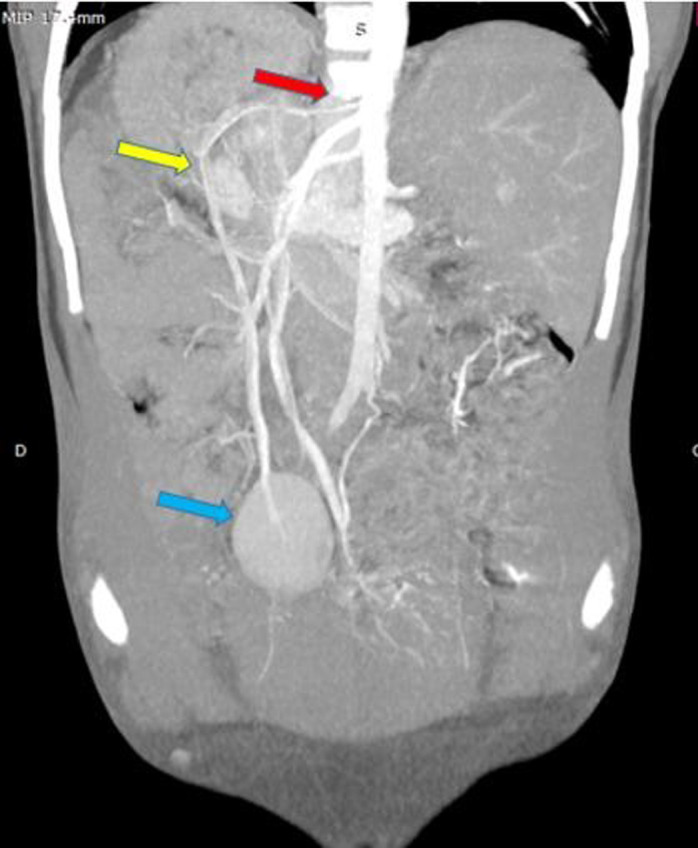
TDM abdominale en coupe coronale (MIP) (10 mm), temps artériel, montrant la vascularisation artérielle (flèche jaune) de la rate baladeuse (flèche bleue) dont l’origine est aortique (flèche rouge)

**Figure 5:**
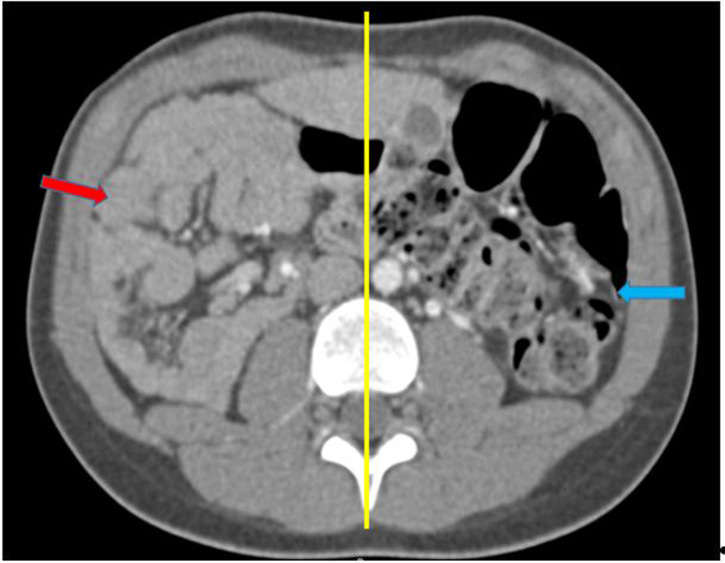
TDM abdominale en coupe axiale, après injection de PDC, montrant un mésentère commun avec l´intestin grêle à droite (flèche rouge) et le colon à gauche (flèche bleue)

Un scanner abdomino-pelvien a été recommandé pour complément de bilan. Cet examen a été réalisé avec un scanner de marque Hitachi^®^Supria mis en service le 1^er^ octobre 2017. Les constantes utilisées étaient de 120 KV et 80 mA et les coupes réalisées étaient de 3 mm jointives. L´examen a été réalisé chez un patient à jeun, en décubitus dorsal, tête en premier. Deux séries d´acquisition ont été effectuées dont l´une sans injection de produit de contraste et l´autre avec injection IV de produit de contraste. Les images acquises après injection IV du produit de contraste ont permis de redresser le diagnostic qui s´établit de la manière suivante: le foie occupait les deux hypochondres avec une taille quasi normale des deux lobes hépatiques ; le hile et la vésicule biliaire étaient sur la ligne médiane (Figure 2, Figure 3). Il n´y avait pas de rate dans les hypochondres et la masse pelvienne sus vésicale était homogène, iso dense au parenchyme splénique et se rehaussait de façon identique à la rate ; c´est la rate en question (il s´agit d´une rate flottante) (Figure 3, Figure 4). Son pédicule vasculaire artériel était directement relié à l´aorte abdominale à hauteur du tronc cœliaque (Figure 4). Une anomalie de rotation du tube digestif à type de mésentère commun complet (de type habituel) avec les anses grêles à droite et le colon à gauche dans l´abdomen a été également notée (Figure 5). Il a été constaté sur les coupes basi-thoraciques obtenues au moment de l´acquisition de la tomodensitométrie (TDM) que le cœur était en position normale à gauche (Figure 3).

**Intervention thérapeutique**: aucun acte d´intervention chirurgical n´est indiqué. Cependant c´est une situation clinique particulière qui impose d´informer le patient sur sa situation particulière. Il a également été sensibilisé sur le fait que d´une la rate en position pelvienne et mobile doit être protégée contre d´éventuel traumatisme et d´autre part à chaque consultation médicale il doit informer le médecin de sa situation particulière. Il a également bénéficié de l´administration d´antalgique (paracétamol) contre les douleurs.

**Suivi et résultats**: en respectant les mesures d´hygiène de vie indiquées et le traitement antalgique, les douleurs abdominales ont été amendées.

**Consentement éclairé**: les parents du patient ont donné leur consentement éclairé pour toute activité scientifique que nous comptons mener sur ce cas.

## Discussion

*Le situs ambigus* est une affection rare, si l'on considère tous les défauts de latéralisation, l'incidence est d'environ 1/10 000 [2] à 1/15 000 et les femmes en sont plus concernées (F>H). *Le situs ambigus* est défini comme étant un défaut de latéralisation des organes, plusieurs tableaux cliniques sont possibles et il peut être associé à n'importe quel type de malformations: cardiaques, rénales, digestives, etc. mais on en retient 2 grands tableaux cliniques: 1) **L´isomérisme droit**: Il s'agirait d'un dédoublement du côté droit; la configuration anatomique thoraco-abdominale des côtés droit et gauche étant identiques à l'image du côté droit par rapport à l´axe du corps, on a donc une duplication des organes situés à droite dans le *situs solitus* et absence possible des organes situés à gauche. Ainsi on a: une asplénie, un foie central, veine cave inférieure à droite et aorte à gauche [3]. 2) **L´isomérisme gauche**: il s'agirait d'un dédoublement de la configuration anatomique du côté gauche; les côtés droit et gauche étant identiques à l'image du côté gauche, on a donc une polysplénie, interruption VCI avec continuation azygos/hemiazygos [3].

*Le situs ambigus* est dans 50 à 100% [4] des cas associés à une malformation cardiaque congénitale. Si le patient ne présente pas de signe de cardiopathie incompatible avec la vie, il peut être asymptomatique, n´étant diagnostiqué qu´accessoirement à l´âge adulte [1], souvent grâce aux examens d´imagerie médicale prescrits pour d´autres plaintes. Très peu d´études ont été consacrées au *situs ambigus* aux vues de son polymorphisme clinique. En Afrique, quelques rares auteurs ont tenu à parler des anomalies de situs [5-7] et du *situs ambigus* en particulier [8]. Il s´agit de la première étude portant sur le *situs ambigus* au Niger.

L´intérêt de notre observation est dans un premier temps son association avec la présence d´une rate flottante ovalaire sus-vésicale. La rate est l´organe lymphoïde le plus large du corps humain, elle apparait vers la 6^e^ semaine à partir de plusieurs foyers tissulaires (précordium splénique) qui fusionnent et déterminent un épaississement de l´épithélium cœlomique du mésogastre dorsal. Anatomiquement elle se situe au niveau de l´hypochondre gauche et présente des contacts avec le diaphragme, le rein gauche, l´estomac, l´angle splénique du colon et la queue du pancréas lui donnant sa forme souvent tétraédrique [9]. Appendue à l´arborisation terminale de l´artère splénique, la rate est maintenue par: les ligaments gastro splénique, spléno-rénal, et phrénico-colique. En cas de déficience de ces ligaments, la rate peut se mobiliser dans l´abdomen (rate baladeuse) et de ce fait il existe un risque de torsion responsable de douleurs abdomino-pelvienne et palpation d´une masse abdomino-pelvienne. La rate baladeuse est une anomalie rare congénitale ou acquise avec une faible prévalence (0,2%) souvent retrouvée chez l´enfant [10]. La prédominance féminine est rapportée dans la population adulte [11,12] mais des cas de sexe masculin ont été rapportés [13]. Le plus souvent, cette anomalie est de découverte fortuite souvent lors d´un examen d´imagerie mais peut également se manifester par des douleurs abdominales comme ce fut le cas chez notre patient [14]. L´association de ce tableau de douleurs abdominales et de masse abdominale mobile doit faire évoquer le diagnostic de torsion de rate baladeuse et faire indiquer un examen d´imagerie pour confirmer le diagnostic [12]; bien que notre cas fût atypique de par la localisation pelvienne d´une masse sensible. L´échographie abdominale constitue l´examen de première intention [12]. Elle permet d´affirmer le diagnostic devant la vacuité de la loge splénique et la mise en évidence d´une masse abdominale rappelant l´écho structure splénique mais dans notre observation le lobe gauche du foie qui occupait les 2 hypochondres nous a fait croire en la présence de la rate dans la loge splénique. Le recours à la TDM a été nécessaire pour redresser le diagnostic. En effet la TDM est la modalité de choix pour le diagnostic de rate baladeuse, surtout lorsqu´une torsion du pédicule est suspectée ou si l´échographie n´est pas très contributive [12].

Le second intérêt de notre observation est l´association à un mésentère commun. Au cours du développement embryologique, le tube digestif subit des phénomènes complexes de réintégration, rotation et accolement. Lorsque ces phénomènes sont incomplets ou vicieux, ils peuvent aboutir à des situations anatomiques potentiellement pathologiques. En occurrence, les anomalies de migration du mésentère: absence totale de rotation, mésentère commun complet, mésentère commun incomplet et rotation inverse en cas de *situs inversus*. Embryologiquement, la première rotation se déroule avant la 10^e^ semaine de gestation lorsque l´intestin primitif est encore situé hors de l´abdomen. Cette rotation place la portion prévitelline (grêle) à droite et la portion post vitelline (colon) à gauche; un arrêt à ce stade est à l´origine du mésentère commun complet. Le mésentère commun complet résulte alors d´un arrêt de la rotation intestinale à 90°. Ainsi se situe, le cadre colique à gauche et intestin grêle à droite; le caecum en position antérieure et médiane et l´artère mésentérique supérieure à droite de la veine mésentérique supérieure [15]. Ce qui fait la particularité de notre observation qui associe un mésentère commun complet à un isomérisme droit. Selon certains auteurs, l'échographie serait l'examen de référence pour éliminer une malrotation intestinale mais celle-ci était non spécifique chez notre patient, ainsi que dans d´autres observations [16,17]. L´apport de la TDM a été donc essentiel pour le diagnostic.

## Conclusion

Le *situs ambigus* constitue une véritable curiosité anatomique de part ces nombreuses présentations cliniques et surtout radiologiques. L´échographie nous a permis de découvrir ces anomalies de position de viscères et la TDM a permis de redresser le diagnostic et de préciser l´anomalie congénitale de positionnement du mésentère. **Perspectives**: les parents du patient avaient été informée sur l´anatomie toute particulière de l´enfant et de l´intérêt de protéger cette rate flottante contre tout traumatisme.
